# In vivo visualization and quantification of collecting lymphatic vessel contractility using near-infrared imaging

**DOI:** 10.1038/srep22930

**Published:** 2016-03-10

**Authors:** Chloé Chong, Felix Scholkmann, Samia B. Bachmann, Paola Luciani, Jean-Christophe Leroux, Michael Detmar, Steven T. Proulx

**Affiliations:** 1 Institute of Pharmaceutical Sciences, Swiss Federal Institute of Technology, ETH Zurich, 8093 Zurich, Switzerland; 2 Biomedical Optics Research Laboratory (BORL), Department of Neonatology, University Hospital Zurich, University of Zurich, 8091 Zurich, Switzerland

## Abstract

Techniques to image lymphatic vessel function in either animal models or in the clinic are limited. In particular, imaging methods that can provide robust outcome measures for collecting lymphatic vessel function are sorely needed. In this study, we aimed to develop a method to visualize and quantify collecting lymphatic vessel function in mice, and to establish an *in vivo* system for evaluation of contractile agonists and antagonists using near-infrared fluorescence imaging. The flank collecting lymphatic vessel in mice was exposed using a surgical technique and a near-infrared tracer was infused into the inguinal lymph node. Collecting lymphatic vessel contractility and valve function could be easily visualized after the infusion. A diameter tracking method was established and the diameter of the vessel was found to closely correlate to near-infrared fluorescence signal. Phasic contractility measures of frequency and amplitude were established using an automated algorithm. The methods were validated by tracking the vessel response to topical application of a contractile agonist, prostaglandin F2α, and by demonstrating the potential of the technique for non-invasive evaluation of modifiers of lymphatic function. These new methods will enable high-resolution imaging and quantification of collecting lymphatic vessel function in animal models and may have future clinical applications.

The lymphatic vasculature is responsible for the maintenance of tissue fluid balance and acts as a conduit for immune cell trafficking and uptake of dietary fat in the intestine. With the advent of specific markers for lymphatic endothelial cells and the discovery of genes responsible for lymphatic specification and growth, the interest in the lymphatic system has rapidly grown^1^. Lymphatic vessels have now been implicated in the pathogenesis of several diseases, including the spread of metastatic tumor cells, chronic inflammation and transplant rejection^2^.

The lymphatic system is comprised of a network of initial vessels that are responsible for the uptake of fluid and macromolecules from the interstitial tissue and collecting vessels that transport lymph fluid through lymph nodes to junctions with the venous system. While initial vessels lack an active pumping mechanism, collecting lymphatic vessels (CLVs) are endowed with a layer of smooth muscle cells that spontaneously contract to force fluid against an increasing pressure gradient through a system of one-way valves^3^. As of yet, there is no established non-invasive imaging approach that can quantify CLV contractility in preclinical models or humans. Such a method could be very valuable to monitor patients with lymphedema or chronic inflammation and to demonstrate effects of therapeutic interventions to increase lymphatic function. In the clinic, magnetic resonance imaging lymphangiography and lymphoscintigraphy with radiotracers are commonly performed to assess lymphatic drainage; however, neither method provides the spatial and temporal resolution required to quantify CLV contractility^4^. Recent efforts have focused on near-infrared (NIR) fluorescent imaging as an alternative. It has been shown that NIR imaging can visualize CLVs in a non-invasive manner in both humans and rodent models^5^,^6^,^7^,^8^.

Accurate estimation of CLV contractility necessitates methods of quantification of changes in vessel tone as well as phasic (spontaneous) contraction frequency and amplitude. Therefore, quantification of lymphatic contractility in animal models has required either invasive *in situ* approaches or *ex vivo* methods that allow direct measurements of vessel diameter^9^,^10^,^11^. *In vivo*, the frequency of contractions has been quantified using non-invasive imaging in several previous studies using either manual counting or semi-automated measures^8^,^12^,^13^,^14^. Non-invasive estimations of vessel tone or contraction amplitude however have been more challenging due to the scattering of photons from overlying skin and other tissue. We hypothesized that NIR fluorescent imaging could be used to estimate both the amplitude (or strength) and frequency of spontaneous lymphatic contractions. Such a system could allow non-invasive quantification of lymphatic contractility after intradermal injection of near-infrared tracers with potential translation to the clinic.

In this study, we aimed to establish an *in vivo* approach allowing the direct visualization and quantification of both the diameter and the fluorescent changes during lymphatic contractility. We developed automated algorithms to quantify assessments of lymphatic contractility, using either diameter tracking or fluorescence imaging data and demonstrate a close correlation between these two measures. The methods were then validated by showing a dose-dependent response in contractility to prostaglandin F2α (PGF2α) applied directly onto CLVs *in vivo*. Finally, the methods were successfully demonstrated non-invasively by measuring the effect of topical skin administration of a nitric oxide (NO) donor on the underlying collecting lymphatic vessel contractility. These results reveal that CLV contractile function and the response of these vessels to contractile modifiers can be imaged and quantified in mice using NIR fluorescent imaging.

## Results

### NIR tracer infusion allows visualization of CLV contractility

**Figure 1:**
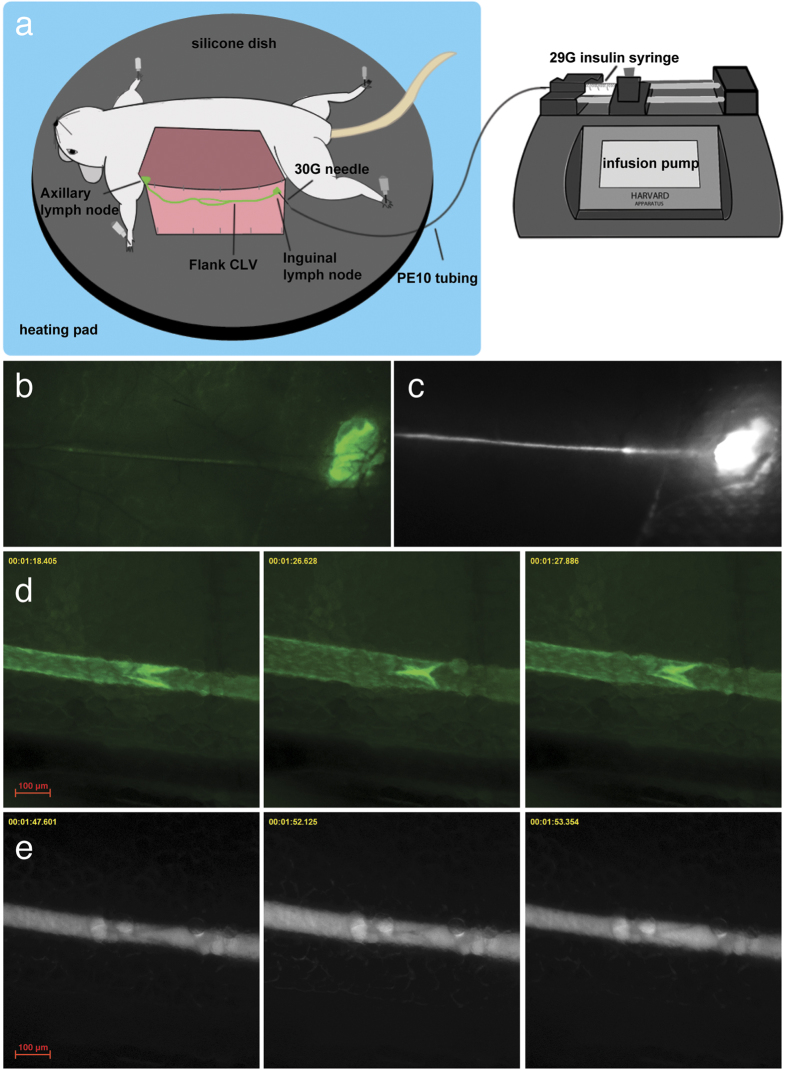
Visualization of a contractile CLV in mice after inguinal lymph node infusion of P20D680. (**a**) Schematic of setup for inguinal lymph node infusion. (**b**) Prox1-GFP image of inguinal lymph node and efferent CLV. (**c**) NIR image after lymph node infusion with efferent lymphatic vessel perfused with P20D680. (**d**) Prox1-GFP signal of stages of contraction cycle of CLV at 63× magnification. (**e**) NIR signal of stages of contraction cycle at 63×.

We first aimed to identify a suitable area that would allow a direct visualization of CLV function. The existence of a contractile CLV under the flank skin of mice that connects the inguinal (subiliac) lymph node to the axillary lymph node has been previously described^8^,^14^,^15^. To allow a more precise injection of lymphatic tracers, we implemented an infusion pump and catheter system for infusion of lymphatic-specific tracer directly into the inguinal lymph node (Fig. 1a). After surgical preparation of Prox1-GFP mice, both the lymph node and the efferent lymphatic vessel were easily visualized with a fluorescence stereomicroscope, using the lymphatic-specific GFP expression to allow easy guidance for catheter implantation (Fig. 1b). After optimization of this procedure, we found that a bolus infusion over 15 s of 500 nL of 10 μmol/L P20D680 lymphatic tracer into the lymph node led to consistent and reliable perfusion of the efferent CLV with no evident leakage from the vessel (Fig. 1c, Supplementary Movie 1).

In Prox1-GFP mice, the lymphatic valves were readily identified by their increased GFP expression. As seen in movies taken before and after the infusion of tracer into the lymph node, the perfusion of tracer led to an apparent increase in the strength of contraction of the CLV and a consistent activation of the valve function (Supplementary Movie 2). High magnification acquisition (Supplementary Movie 3) showed the valve opening during the contraction of the upstream lymphangion and closing shortly after the contraction of the downstream lymphangion, consistent with previous descriptions of lymphatic contractility and valve function *ex vivo*^3^. The phases of one contraction cycle with visualization by the Prox1-GFP and the NIR signals are shown in Fig. 1d,e, respectively.

### NIR signals allow accurate diameter tracking of CLVs

**Figure 2:**
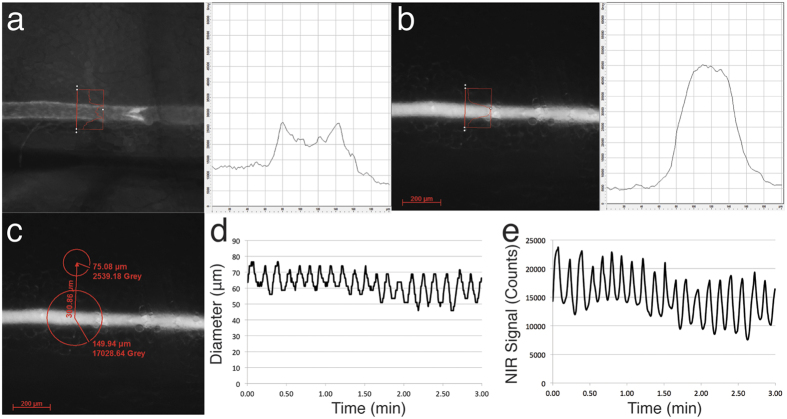
Diameter tracking of CLVs. (**a**) Signal profile of line bisecting a Prox1-GFP CLV. (**b**) Signal profile of line bisecting a P20D680 perfused CLV. (**c**) ROI analysis to determine appropriate threshold for CLV diameter tracking using NIR signals. (**d**) Contractility plot demonstrating results from diameter tracking using NIR signals of a CLV for a 3-min movie. (**e**) Corresponding contractility plot demonstrating fluorescent signal from region of interest over vessel.

Measurements of lymphatic contractility have relied on accurate tracking of lymphatic diameters during the contraction cycle. These measurements are typically performed using phase-contrast imaging of lymphatic vessels and depend on either manual or automated analysis algorithms to track the diameter of these vessels^16^,^17^. We aimed to develop a simplified method for quantification of the diameter, using the fluorescent signals from either Prox1-GFP or NIR imaging after injection of P20D680. We first evaluated the fluorescent signal profile of a line bisecting the CLV using either GFP (Fig. 2a) or NIR (Fig. 2b) imaging. As seen in Fig. 2a, the edge of the GFP^+^ lymphatic vessel displayed an approximate 2-fold signal increase compared to the surrounding tissue. This profile then declined in signal within the lumen of the vessel and increased again at the other edge. By comparison, the signals from the NIR imaging displayed a much higher signal (typically >6-fold above background signals) and the profile was more consistent over the entire vessel width (Supplementary Movie 4). In addition, since the NIR tracer was only present within the vessel, the signal represents the inner diameter and more accurately reflects the pumping of lymph fluid.

Based on this observation, we developed a simple algorithm that tracks the diameter of the lymphatic vessel over time, using the vessel profile data from NIR imaging. We first established a proper threshold to define the vessel signal from the surrounding tissue. Since there are differences in perfusion of tracer and due to the contractility itself, the NIR signal within the vessel does not always have the same intensity as compared to the tissue background. Therefore, we employed a “moving threshold” based on the signal at each time point from a region of interest (ROI) placed over the vessel normalized to the background signal from an ROI placed at a measured distance from the vessel (Fig. 2c). Then, using the signal for each pixel in a line profile normalized to the background signal, we calculated the number of pixels above this threshold and therefore the diameter of the vessel at each time point in the acquisition (Fig. 2d). We validated our diameter measurements by comparing them to manual measures of diameter at 10 random time points per vessel and found close agreement (mean absolute deviation =3.76 μm, root square mean deviation =4.62 μm, n = 15 vessels).

Comparison of the plots of vessel diameter over time (Fig. 2d) with the fluorescent signal over time (Fig. 2e) showed excellent overlap. We found that the diameter plots were not as smooth in nature when compared to fluorescence plots, which was due to the relatively poor spatial resolution (512 × 512 pixels) of the highly NIR sensitive EMCCD camera. However, data from movies acquired of the lymphatic contractility demonstrated highly significant correlations between the diameter measurements and the fluorescence data (n = 15 mice, p < 0.001 in each case) with a mean Pearson r correlation value of 0.902 ± 0.054. These data indicate that the fluorescent signals themselves may be an accurate reflection of the contraction cycles of the CLV.

### Automated quantification of lymphatic contractile parameters

**Figure 3:**
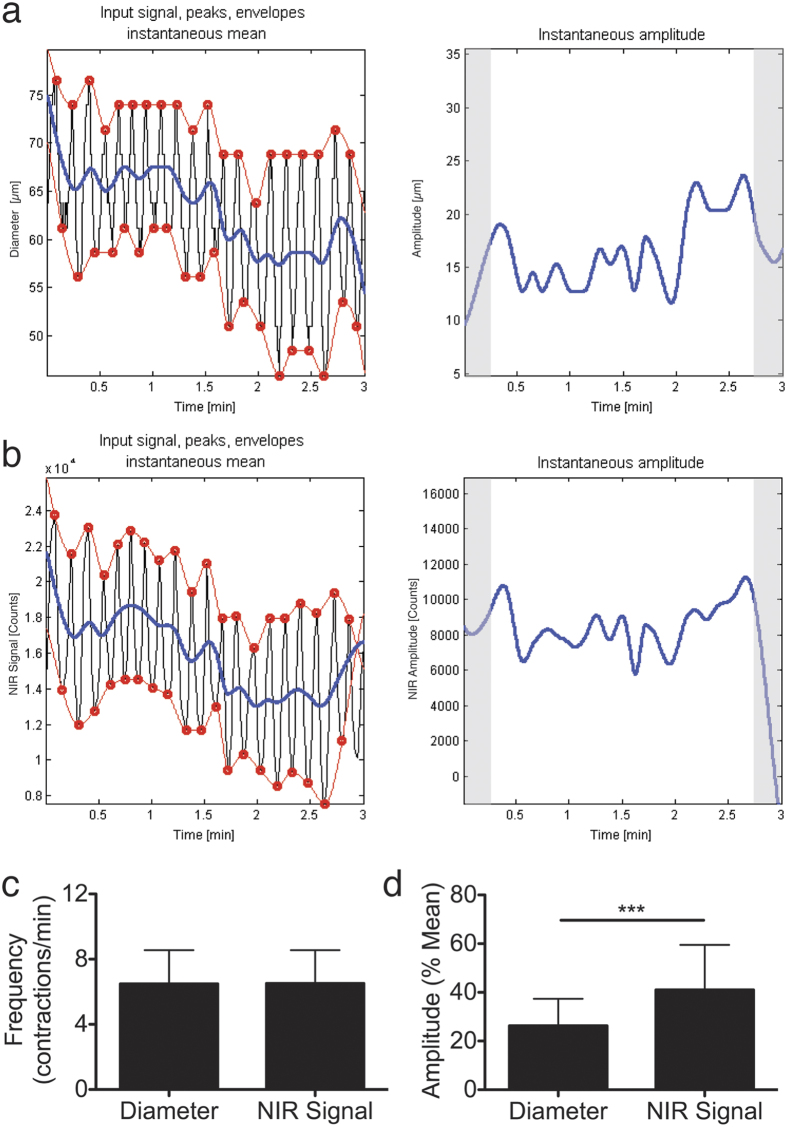
Automated assessments of frequency and amplitude from contractility plots of diameter or fluorescence data. (**a**) Visual output of Matlab algorithm using diameter tracking data to determine peaks and troughs (red circles), instantaneous mean (blue line in left plot) and instantaneous amplitude (blue line in right plot). Shaded regions represent data that are excluded from the quantification but are necessary for the algorithm to calibrate. (**b**) Visual output of Matlab algorithm using NIR signal data. (**c**) Quantification of frequency in contractions per min. (**d**) Quantification of amplitude expressed as percent of instantaneous mean. n = 15 mice, ***represents P < 0.001 (two-tailed Student’s *t*-test). Data are mean ± SD.

We next aimed to develop an automated system to quantify lymphatic contractile parameters by detection of the peaks and valleys of the diameter and NIR signal vs. time plots. This would allow a simplified quantification of the frequency of contractions and the amplitude of each contraction cycle. We adapted an algorithm originally developed for detection of peaks in an oscillatory pattern^18^. The algorithm detects peaks and troughs of the pattern and calculates the mean fluorescence intensity over time. The frequency of contractions is calculated by counting the peaks per min. The amplitude of the contractions then is calculated as a function of % change from the mean NIR signal intensity or diameter to adjust for differences in brightness or vessel size, respectively (Fig. 3a,b).

When comparing the output of our algorithm using both sources of data, we again found close correlation of diameter and fluorescence data. Frequency results from the fluorescence and diameter data were essentially identical with 6.51 ± 2.05 and 6.49 ± 2.07 contractions per min, respectively (Fig. 3c). Interestingly, we found that the % amplitude was of significantly higher magnitude when calculated from the fluorescence data as compared to the % amplitude from the diameter tracking (41.01 ± 18.46% vs. 26.27 ± 11.08%, p < 0.0001) (Fig. 3d). This likely implies that the NIR fluorescence data are more indicative of volume changes within the vessel than the one-dimensional assessment of diameter. Since the efficiency of phasic contractility depends on both frequency and amplitude, we have devised a simple measure of pumping efficiency as frequency x % NIR signal amplitude. The mean pumping score of the flank collector vessels was 262.44 ± 126.54.

### Prostaglandin F2α stimulates CLV contractility

**Figure 4:**
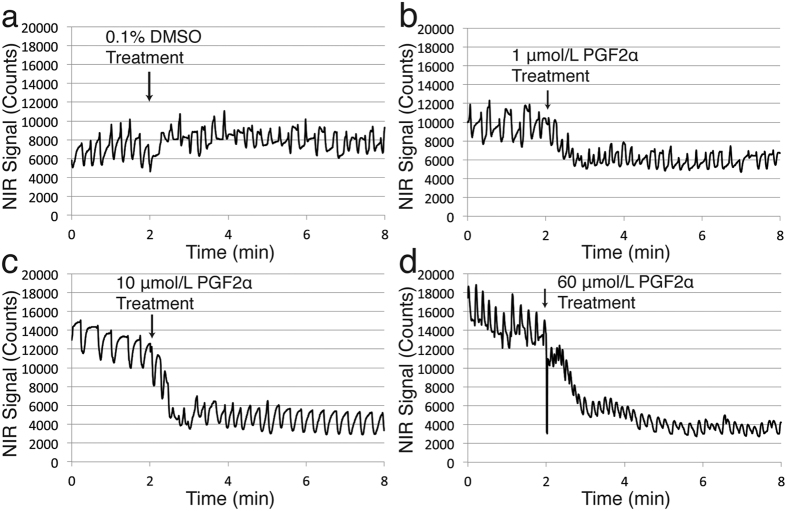
Representative NIR signal contractility plots of CLV response to topical treatment of PGF2α. Eight-min movies were acquired with administration of 40 μL of either 0.1% DMSO vehicle control or 1 μmol/L, 10 μmol/L or 60 μmol/L PGF2α at t = 2 min. (**a**) Response to 0.1% DMSO. (**b**) Response to 1 μmol/L PGF2α. (**c**) Response to 10 μmol/L PGF2α. (**d**) Response to 60 μmol/L PGF2α.

We next tested the ability of this imaging setup to visualize and quantify the effect of an acute application of the known contractile agonist PGF2α on CLV function^19^,^20^. After infusion of P20D680 and verification of steady contractile function, we initiated an 8-min movie. At 2 min into these movies, we added 40 μL of either 0.1% DMSO (as vehicle control) or a range of concentrations of PGF2α (1, 10 and 60 μmol/L) topically onto the CLV. Application of 0.1% DMSO showed no major effects on contractility when comparing the fluorescent signals of the CLV before and after treatment application (Fig. 4a, Supplementary Movie 5). A low concentration of PGF2α (1 μmol/L) led to slight phasic effects with most vessels responding with increased frequency (Fig. 4b, Supplementary Movie 6). For 10 μmol/L PGF2α, a tonic effect was observed on the CLV immediately after application with a concurrent phasic effect of increased contraction frequency (Fig. 4c, Supplementary Movie 7). Despite this increase in tone, the raw amplitude of contractions was more or less maintained. A higher (60 μmol/L) concentration of PGF2α led to strong tonic effects with increased contraction frequency and decreased raw amplitude (Fig. 4d, Supplementary Movie 8).

**Figure 5:**
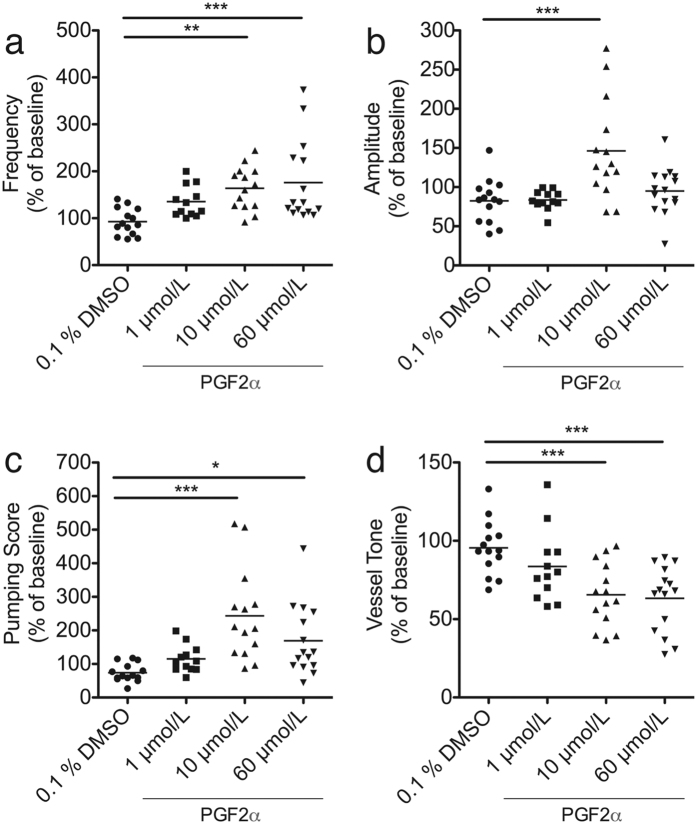
Quantifications from NIR signal contractility plots of CLV response to topical treatment of PGF2α. Quantifications from contractility plots from the post treatment period of t = 3 to 6 min were normalized to the baseline data from t = 0 to 2 min and expressed as a % of baseline. (**a**) Frequency of contractions. (**b**) Percent amplitude of contractions. (**c**) Pumping score (frequency x % amplitude). (**d**) Vessel tone. Number of vessels analyzed: n = 14 DMSO, n = 12 1 μmol/L PGF2α, n = 14 10 μmol/L PGF2α, n = 15 60 μmol/L PGF2α. *P < 0.05, **P < 0.01, ***P < 0.001 (one-way ANOVA with Dunnett’s multiple comparison test).

We quantified these responses by comparing the PGF2α treatment effects to 0.1% DMSO. Contractile parameters were derived from the fluorescent signals of the vessel at baseline (0 to 2 min) and post-treatment (3 to 6 min corresponding to 1 to 4 min after treatment). To study the changes in contractile parameters due to treatment, post-treatment values were compared to baseline measurements normalized to 100%. The results indicated a dose-dependent rise in contraction frequency (Fig. 5a) for PGF2α (1 μmol/L PGF2α: 135.3% ± 33.21%, p = 0.156; 10 μmol/L PGFa: 163.7% ± 46%, p < 0.01; 60 μmol/L PGF2α: 175.8% ± 86.85% p < 0.001) when compared to 0.1% DMSO (92.62% ± 28.57%). The % amplitude of the contractions (Fig. 5b) was not significantly different from 0.1% DMSO (82.46% ± 28.11%) for all concentrations except for 10 μmol/L PGF2α (146.2% ± 63.7%, p < 0.001). As a result, the pumping score (Fig. 5c) was significantly increased for 10 μmol/L PGF2α (243.4% ± 136.6%, p < 0.001) and, to a lesser extent, also for 60 μmol/L PGF2α (169.2% ± 105.3%, p < 0.05) compared to 0.1% DMSO (73.75% ± 26.9%). A tonic effect in response to higher concentrations of PGF2α was also observed when compared to 0.1% DMSO (95.47% ± 17.33%). This increased tone was significant for 10 μmol/L PGF2α (65.61% ± 20.24%, p < 0.001) and 60 μmol/L PGF2α (63.27% ± 20.86%, p < 0.001) (Fig. 5d).

Of note, when quiescent vessels were occasionally observed after the infusion of P20D680, they were also able to be activated and began contracting after addition of PGF2α (Supplementary Movie 9). Importantly, we were able to validate the effects of PGF2α to stimulate lymphatic contractility using non-perfused vessels in Prox1-GFP mice. DMSO again showed no major effect (Supplementary Movie 10) while PGF2α increased the tone and the frequency of contractions (Supplementary Movie 11). Consistent activation of valve function was also visualized with addition of PGF2α. Together, these results show that the developed methods using NIR imaging are able to quantify effects of topically administered agents on lymphatic contractility in mice *in vivo*.

### Non-invasive assessment of a NO donor on CLV contractility

**Figure 6:**
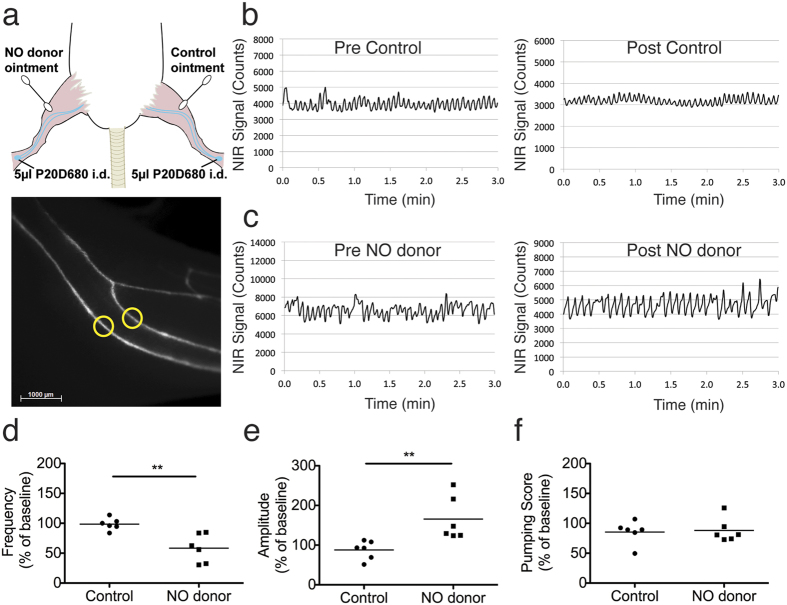
Non-invasive imaging of CLV contractility response to skin treatment of NO donor. (**a**) Schematic indicating experimental design and a representative picture from non-invasive imaging of CLVs. Each mouse served as its own control with the NO donor (Rectogesic) treatment applied to the left leg and then the control (Linola fett) treatment applied to the right leg. Contractility was assessed in two vessels per leg (yellow ROIs). (**b**) Representative contractility plots from pre- and post-control treatment conditions. (**c**) Representative contractility plots from pre- and post-NO donor treatment conditions. The Y-axis scale is adjusted in each case such that the mean fluorescent intensity values fall in the center of the axis, in order to better visualize differences in percent amplitude. Quantifications from contractility plots from the post-treatment period were normalized to the pre-treatment data and expressed as a % of baseline. Raw values can be found in Table 1. (**d**) Frequency of contractions (**e**) Percent amplitude of contractions. (**f**) Pumping score (frequency x % amplitude). n = 6 C57BL/6J-*Tyr*^*c-J*^ albino mice were analyzed. *P < 0.05, **P < 0.01, ***P < 0.001 (two-tailed Student’s *t*-test).

Finally, we aimed to demonstrate that the developed methods could be applied to non-invasive conditions. For these experiments, we tested the effect of a skin-applied NO donor, rectogesic, which contains glyceryl trinitrate and has been shown to reduce lymphatic transport in both humans and rodent models^7^,^21^. Although imaging of the flank CLV is possible in a non-invasive manner in mouse models^8^,^12^,^14^, we have found that the CLVs leading to the popliteal lymph node in mice can be perfused more consistently after intradermal injection into the dorsal aspect of the hind paw. Therefore, we tested the effects of NO donor administration in comparison to a control ointment on the contractility of the popliteal CLVs (Fig. 6a). After removal of the fur in the lower limbs we injected 5 μL of P20D680 into the paw skin and acquired NIR videos to assess the baseline contractility of the CLVs between the ankle and popliteal region (Fig. 6b,c). As shown in Table 1, there were no major differences in the baseline CLV contractility measurements between the NO donor treated limbs and the control treated limbs. Next, we applied a thin layer of either rectogesic or control ointment to the skin and after 3 min acquired a second NIR video for contractility assessment (Fig. 6b,c, Supplementary Movie 12). By calculating the percent change from the baseline condition we were able to demonstrate that the NO donor significantly reduced the frequency of CLV contractions in comparison to control (58.41% ± 23.78% vs. 98.45% ± 10.02%, p < 0.01, Fig. 6d). However, the NO donor treatment led to a significant increase in the amplitude of CLV contractions compared to control (165.63% ± 54.95% vs. 87.87% ± 23.43%, p < 0.01, Fig. 6e). This led to no significant difference in the percent change of the pumping score between the two groups (88.12% ± 19.94% vs. 85.39% ± 19.13%, p = 0.81, Fig. 6f).

## Discussion

In this study, we have developed a novel assay to visualize and sensitively measure murine CLV contractility using stereomicroscopic imaging. We have demonstrated the suitability of this method to visualize valve function and tonic and phasic changes in lymphatic contractility. Semi-automated assessments of vessel diameter and lymphatic contraction frequency and amplitude were established and validated using topical administration of a contractile agonist. The methods were also extended to the non-invasive situation by testing the effect of a skin-applied NO donor on the contractility of collecting lymphatic vessels located under the skin.

Mesenteric lymphatic vessels are the most common CLV assayed for contractile function in species such as cow, guinea pig and rat, using both isolated CLVs and *in vivo* preparations^10^,^19^,^22^,^23^. Although there has been one previous report demonstrating the contractility of mesenteric lymphatic vessels in the DDY strain of mice^24^, it is generally considered that mouse mesenteric vessels in most strains do not show consistent contractility. The contractility of popliteal CLVs in mice has been investigated in a previous *in vivo* assay in which FITC-dextran was injected into the paw and the vessels were imaged by inverted microscopy after surgical intervention^11^. While we also confirmed that popliteal CLVs can be evaluated for contractility in a non-invasive assay after intradermal injection of NIR lymphatic tracers^8^, we found that after removal of skin, these vessels were typically buried deep in fat and when exposed seemed to lose their contractile behavior (unpublished observations). Therefore, we aimed to identify a new anatomical location to allow direct visualization and analysis of lymphatic contractility. We found that the flank skin of the mouse allowed easy surgical access as well as a reliable site for introduction of tracers directly into the lymphatic system using infusion into the inguinal lymph node. Additionally, the flank vessels exhibit extensive smooth muscle coverage with strong consistent contractility^8^,^14^. Another advantage of this location is that the direct exposure of this vessel allows for evaluation during topical administration of pharmacological agents.

After exposure of the inside of the flank skin, Prox1-GFP mice allowed for high resolution imaging of CLV function and valve function. A potential advantage of imaging the CLV function with a fluorescent reporter mouse strain is that it does not require an infusion of tracer. However, as Prox1-GFP signal is highly expressed in the nuclei of the endothelial cells within the wall of the vessels, the signal visualized was not ideal for quantifying the vessel inner diameter, which is necessary for accurate estimation of pumped lymphatic fluid. The high background of the surrounding tissue from autofluorescence in the green region of the visible spectrum also complicated the quantitative analysis necessary to measure diameter over time. We found that imaging in the NIR range after perfusion of P20D680 improved the visualization of the vessel compared to background, allowing for accurate diameter tracking after employing a simple threshold method for vessel edge detection. NIR signals from CLVs could also be assessed by a ROI analysis, which can be performed through skin after intradermal injection of tracers, enabling non-invasive quantification^8^. Previous quantifications using NIR fluorescence imaging have focused on quantifying the frequency and/or velocity of ICG packets or pulse rates over time^7^,^8^,^12^,^13^. Importantly, our demonstration of a close correlation between diameter tracking and NIR fluorescence signals indicates that more robust contractile function measurements of both frequency and amplitude may now be possible in a non-invasive manner, potentially allowing quantification of CLV contractility in humans using NIR imaging^4^,^5^,^6^. A reduction of CLV pumping strength is associated with the development of secondary lymphedema, a finding that was observed even at early stages of the disease, therefore improved quantitative NIR imaging techniques may lead to improved detection and the ability to monitor the response to therapeutic interventions in lymphedema patients^25^.

We devised an algorithm that can detect peaks and troughs of contractility plots to provide automated measurements of contraction frequency and amplitude. We then compared the assessments of amplitude derived from diameter measurements and fluorescence measurements and found that the % amplitude was significantly greater from the fluorescence measurements. Typically, to quantify a lymphatic “stroke volume”, investigators have modeled the vessel as a cylinder and calculated the cross sectional area changes from the diameter values^10^. With fluorescence data, this may no longer be necessary, as the % amplitude itself may provide stroke volume information. We have developed a pumping score that is a product of the frequency and % NIR signal amplitude (as an estimate of strength of contraction) as a measure of collecting vessel phasic contractility.

We next tested the ability of our imaging preparation and quantitative measures to detect changes in contractility to a known contractile agonist of CLVs, PGF2α^19^,^20^,^26^. We found that acute application of PGF2α onto exposed CLVs resulted in both tonic and phasic effects on lymphatic contractility. At concentrations of PGF2α above 10 μmol/L, there was a strong acute tonic response with maximum constriction of the vessel observed at 1 to 2 min after application of the agonist. This tonic effect could be quantified as the mean fluorescence intensity change before and after application since as the vessel constricted, the volume of lymph fluid and tracer within the vessel was reduced. We also detected immediate agonistic effects on phasic contractility with maximum responses in frequency and % amplitude at 10 μmol/L PGF2α, leading to a significantly increased pumping score. Importantly, by use of Prox1-GFP mice, we were able to demonstrate that these responses occurred whether or not the lymphatic vessel has been perfused with tracer. Our results are consistent with the contractile effect of PGF2α that has been demonstrated in several studies using isolated lymph segments of both bovine and human origin^19^,^20^,^26^,^27^. A PGF2α analogue has been suggested as a potential therapy for lymphedema due to its contractility effects as well as reduction of lymphatic capillary pressure^28^,^29^. PGF2α analogues, such as latanoprost, are also used as a leading therapy for glaucoma, a disease that the lymphatic system has recently been implicated to play a role^30^,^31^,^32^.

We also demonstrated the ability of the new quantitative techniques to measure CLV contractility in a non-invasive manner, an important step for possible clinical translation. Towards this end, we evaluated the effect of a NO donor, comprised of glyceryl trinitrate, on the collecting lymphatic vessels after application to the skin of the lower limb of the mice. The formulation has previously been demonstrated *in vivo* to impair lymphatic transport of radiotracers and snake venom in humans and rats, respectively^21^, and has been shown to reduce lymphatic transport of indocyanine green in a rat tail model^7^. We showed that the addition of NO led to decreased frequency of contractions compared to the control ointment consistent with many previous reports of NO effects on CLV contractility^7^,^11^,^23^,^33^,^34^,^35^. However, surprisingly, application of the NO donor led to a CLV response with increased contraction amplitude and an overall effect on the pumping score that was not significantly different than the control ointment group. There are discrepancies in the literature regarding the effect of NO on CLV amplitude or tone. While some authors report phasic contractility effects of decreased amplitude^33^,^34^, others have published results that align more closely with ours^35^,^36^. In particular, a study by Gasheva *et al.* demonstrated that addition of an NO inhibitor, L-NAME, to isolated rat thoracic ducts led to contractile response of increased frequency and decreased amplitude^35^. These results led to a consensus that there may be biphasic responses to NO by CLVs with high doses leading to decreases in frequency and amplitude (allowing the vessel to act more like a passive conduit for flow) and lower doses leading to a positive lusitropic effect (with the reduced frequency allowing more time for vessel filling and, hence, stronger contractions). However, more recently, an extensive study using isolated murine popliteal collecting lymphatic vessels, the same vessel we have evaluated *in vivo*, concluded that the major effect of NO was through an decrease in tone with no effects on phasic contraction amplitude^37^. Unfortunately, we could not accurately measure changes in lymphatic tone in the present experiment since there was interference from the topically applied cream to the skin that affected the acquired fluorescent signals. In sum, it appears that more research is necessary to further elucidate the role of NO on lymphatic contractility.

There are some limitations of the *in vivo* contractility assays. As we have shown, infusion of tracer led to an activation of the vessel and valve function, most probably due to a transmural pressure increase^22^. This may complicate the subsequent analysis of contractile factors; however, in this study, we were still able to demonstrate the ability of an agonist to stimulate the vessels. It is important to allow the system to stabilize after infusion, as well as to use non-perfused fluorescent reporter mice as a further control to test compounds under more physiological conditions. Surgery and deep anesthesia may also effect contractility; however, we found contraction frequencies in a similar range for the flank CLV as previously reported by others using a non-invasive approach^14^. Additionally, with *in vivo* assays, exact pressures and flow rates cannot be controlled unlike in *ex vivo* perfusion approaches. Despite this, an *in vivo* approach has advantages over preparations that involve isolated lymphatic vessel segments. The dissection process to isolate *ex vivo* lymphatic vessels will sever connections to the nervous system and may disrupt the fragile endothelial cell lining^38^. Since both cell types are known to influence smooth muscle cell contractility^39^, it is likely that an *in vivo* system will enable more physiologically relevant responses to pharmacological administrations. The current techniques likely will complement existing *ex vivo* approaches, including those recently established using isolated mouse lymphatic tissue^37^,^40^, for evaluation of lymphatic function in transgenic animals and to test responses to novel therapeutic interventions. Additionally, the ability to monitor and quantify collecting lymphatic vessel contractility with non-invasive approaches may have clinical potential to evaluate patients with lymphedema and chronic inflammation.

## Methods

### Mice

C57BL/6J-*Tyr*^*c-J*^ albino mice (Jackson Laboratories, Bar Harbor, ME) were maintained under pathogen-free conditions until imaging. Prox1-GFP mice on a C57BL/6J background were a kind gift of Dr. Young-Kwon Hong, University of Southern California^41^. After imaging, the mice were euthanized with an overdose of anesthesia (1000 mg/kg ketamine; 3.5 mg/kg medetomidine) followed by cervical dislocation. All experiments were performed in accordance with animal protocols approved by Kantonales Veterinaeramt Zurich (protocols: 11/2012 and 12/2015).

### Lymphatic-specific imaging tracers

The poly(ethylene glycol) (PEG)-based lymphatic tracer P20D680 was prepared as previously described^8^. Briefly, methoxyPEG amine P20 (20 kDa) was reacted equimolarly with IRDye^®^ 680LT NHS Ester in 500 μL anhydrous dimethyl sulphoxide (DMSO). The crude reaction was then diluted with 4 mL of ultrapure water and freeze-dried overnight to remove completely any trace of organic solvents. The lyophilizate was then reconstituted with 250 μL of HEPES buffered saline (20 mmol/L HEPES, 145 mmol/L NaCl, pH 7.4) and purified. Tracer stock solutions were maintained at −20 °C until further use.

### Stereomicroscopic imaging of flank CLVs

Mice were prepared for surgery via deep anesthesia using a combination of medetomidine (0.8 mg/kg, Domitor®, Provet, Lyssach, Switzerland) and ketamine (140 mg/kg, Ketasol®, Graeub, Bern, Switzerland) administered via an intraperitoneal injection. Regulations for deep anesthesia were altered by the Kantonales Veterinaeramt Zurich in 2014 and therefore a combination of xylazine (20 mg/kg, Rompun® 2%, Provet), ketamine (100 mg/kg, Ketasol®) and acepromazine (3 mg/kg, Prequillan®, Arovet, Dietikon, Switzerland) was administered after this date. No detectable differences in lymphatic contractility with the two anesthesia protocols were observed. After the mice were unresponsive to a toe pinch, the extremities were fixed on a silicone-coated stage using cannulas. A median incision starting at the pubic bone and ending at the upper end of the sternum was performed. After blunt separation of the skin from the peritoneum on one side, two additional cuts were made, one towards the inguinal and the other towards the axillary region, sparing large blood vessels in these regions. The skin flap was fixed on the silicone stage using dissecting pins in order to get a flat surface for imaging and to minimize the movement of the flap due to breathing. To prevent drying out, sterile warmed PBS was administered on the exposed tissues. During the experiment, the mice were maintained on a heating pad to prevent loss of body temperature. In case the imaging time exceeded 75 to 80 min an additional intraperitoneal injection of anesthesia with 1/3 of the initial dose was administered. The total imaging time did not exceed 2 h.

A Zeiss StereoLumar.V12 stereomicroscope with AxioVision (Carl Zeiss, Feldbach, Switzerland) software and a Photometrics Evolve 512 camera (Photometrics, Tuscon, AZ) were utilized for NIR *in vivo* imaging^8^. A custom-designed catheter with polyethylene PE-10 tubing (SCI, Lake Havasu City, AZ) and a 30-g needle was inserted into the inguinal lymph node, and a 15 s infusion of 500 nL of 10 μmol/L P20D680 was made using an infusion pump (PHD2000, Harvard Apparatus, Cambridge, MA). Adequate perfusion of tracer was confirmed, and the vessel was observed for several minutes to ensure that the lymphatic contractility was active and that no obstructions (due to masses of cells or air bubbles) had occurred. Imaging was performed at exposure times of 50 to 200 ms with illumination by LED (coolLED, Andover, UK) at 635 nm and a 50 nm emission bandpass filter centered at 690 nm.

### CLV diameter analysis and automated contractility assessment

The diameters of CLVs over time were tracked in acquired 3-min movies at 2.5 frames/s and 63× magnification. In Axiovision software, we first determined a suitable threshold to define the edges of the perfused vessel by drawing region of interests (ROIs) over the lymphatic vessel and over the background tissue. To do this, we drew a ROI with radius r over the center of the lymphatic vessel. We then drew a line perpendicular to the vessel axis of length 2r from the center of the vessel extending into the surrounding tissue. At the end of this line, we then drew a second ROI of radius 0.5r to define the background signal. The mean signal intensity signals for each ROI over time were then exported as .xml files. The threshold for the vessel edge was defined as the mean signal intensity of the vessel ROI divided by the mean signal intensity of the background ROI.

Next, a second line was drawn from the center of the background ROI extending through the vessel to the far edge of the vessel ROI. The signal intensity for each pixel along this vessel length at each time point was then determined using the Profile function in Axiovision and exported as a table in .xml format. In Microsoft Excel, these data were then normalized to a background signal defined as the mean signal of the first ten pixels of this line at each time point. Then, at each time point, a COUNTIF function was used to count the number of pixels along the line length with a normalized signal intensity that was above the threshold that was defined above using ROIs. Finally, the number of pixels was multiplied by the scaling factor (μm/pixel) to determine the diameter over time.

These data were then analyzed using a custom program written in Matlab (The Mathworks, Natick, MA) for assessments of the frequency and amplitude of the contractility of CLVs based on an automatic multiscale-based peak detection algorithm^18^. The complete Matlab code can be found in the Supplementary Methods.

### Prostaglandin F2α administration on flank collecting vessels

We tested the imaging setup by analyzing the effect of the known contractile agonist of CLVs, PGF2α^26^. For this purpose, PGF2α (Enzo Life Sciences, Farmingdale, NY) was dissolved in DMSO (Sigma-Aldrich, Buchs, Switzerland) at a final concentration of 60 mM, aliquoted and stored at −20 °C. PGF2α was diluted in PBS to the desired concentrations of 1, 10 and 60 μmol/L and kept on ice on the day of use. Final DMSO concentrations did not exceed 0.1% (v/v), which served as vehicle control. The flank CLV was perfused as described above, and after visualization of regular vessel contractions, 8-min movies were taken at 2.5 frames/s and at 63× magnification. At a timepoint of 2 min, the vessel was topically treated with 40 μL of 0.1% DMSO, 1, 10 or 60 μmol/L PGF2α, and vessel contractions were recorded for the next 6 min.

On one exposed flank CLV, we always first applied 0.1% DMSO as vehicle control before analyzing CLV contractility with increasing concentrations of PGF2α. In between movies, flank CLVs were flushed with warm PBS and care was taken that new regions of the vessel were chosen for each PGF2α concentration. Baseline contractility measurements were analyzed using the Matlab algorithm from 0–2 min of the 8-min movies, and contractility changes to treatment at 3–6 min. Tonic changes upon PGF2α treatment were calculated by comparing the mean fluorescence intensity over time for pre- and post-treatment. Frequency, amplitude, pumping score (frequency x % NIR signal amplitude) and tonic changes were plotted as percentage from baseline measurements (0–2 min) of each CLV.

### Non-invasive evaluation of a nitric oxide donor on CLV contractility

Evaluation of hind limb CLV contractility was performed using methods similar to those described in a previous report^8^. Mice (n = 6) were anesthetized and prepared for imaging by removal of fur from the lower hind limbs with a razor and depilation cream. Mice were then positioned under the StereoLumar.V12 stereomicroscope and an intradermal injection of 5 μL of 20 μmol/L P20D680 was made into the dorsal aspect of the left paw. After confirmation of CLV contractility and the stability of the NIR signals, a 3-min video was acquired (200 ms exposure, 2.5 frames/s) for analysis of baseline contractility. Then, a cotton swab was used to apply a thin layer of the NO donor, Rectogesic® (Glyceryl trinitrate 4 mg/g, Cederberg GmbH, Binnigen, Switzerland) to the skin of the lower limb excluding the injection site. Three minutes after application, a second 3 min video was acquired with the same settings as before. After this, a second injection of 5 μL of 20 μmol/L P20D680 was performed into the right paw and baseline video was acquired as before. A control emulsion (Lanola fett, Alcina AG, Muttenz, Switzerland) containing similar base compounds as Rectogesic® (lanolin, vaseline, paraffin and sorbitan) was then spread onto the skin and a second video acquired as above. On each video, two vessels were evaluated using ROI analysis. Baseline and post-treatment contractility measures were determined using the Matlab algorithm and frequency, amplitude and pumping score were plotted as percent change from baseline for each CLV. The values from the two vessels were then averaged to obtain one value for each measure per leg.

### Statistics

Linear correlation analysis was performed using a Pearson correlation to determine r and two-tailed p values. A root square means approach was used to determine the absolute deviation and root square mean deviations of manual measurements versus automated measurements of diameter from 10 random time points per movie. Two-tailed student’s t tests were performed to test between means of two groups, while one-way ANOVA with a Dunnett’s post-hoc test was used to test between means of multiple groups against a control group. P < 0.05 was accepted as statistically significant. Outliers were identified using Grubb’s test (p-value < 0.05) and were excluded from the analysis.

## Additional Information

**How to cite this article**: Chong, C. *et al.* In vivo visualization and quantification of collecting lymphatic vessel contractility using near-infrared imaging. *Sci. Rep.* **6**, 22930; doi: 10.1038/srep22930 (2016).

## Supplementary Material

Supplementary Information

Supplementary Movie 1

Supplementary Movie 2

Supplementary Movie 3

Supplementary Movie 4

Supplementary Movie 5

Supplementary Movie 6

Supplementary Movie 7

Supplementary Movie 8

Supplementary Movie 9

Supplementary Movie 10

Supplementary Movie 11

Supplementary Movie 12

## Figures and Tables

**Table 1:** Non-invasive CLV contractility assessments from control ointment and NO donor groups during pre- and post-treatment conditions.

	Pre-treatment			Post-treatment		
	Frequency(min^−1^)	Amplitude(% of MFI)	Pumping Score	Frequency(min^−1^)	Amplitude(% of MFI)	Pumping Score
Control	12.58 ± 1.30	28.32 ± 13.46	336.67 ± 123.21	12.39 ± 1.72	22.90 ± 6.70	272.53 ± 68.90
NO donor	14.36 ± 1.46	26.13 ± 8.15	362.65 ± 90.40	8.47 ± 3.95	40.61 ± 8.54	313.73 ± 78.50

Values are reported as mean +/− standard deviation.
