# Supplementary material for: In vivo visualization and quantification of collecting lymphatic vessel contractility using near-infrared imaging
Source: Sci Rep. 2016 Mar 10;6:22930. doi: 10.1038/srep22930 (PMC4785392; doi:10.1038/srep22930)
Supplement: Supplementary Information [file srep22930-s1.pdf]

In vivo visualization and quantification of collecting lymphatic vessel contractility using near-infrared imaging

Chloé Chong[1*], Felix Scholkmann[2*], Samia B. Bachmann[1], Paola Luciani[1], Jean-Christophe Leroux[1], Michael Detmar[1] and Steven T. Proulx[1]

Supplementary Material

Supplementary Methods

Matlab software code for automated analysis of contractile parameters of CLVs

The Matlab code enables the identification of peaks and troughs in the recorded time series by using an automatic multiscale-based peak detection (AMPD) algorithm [1]. AMPD performs an automatic peak detection of non-stationary multiscale time series based on the analysis of the local maxima scalogram, a matrix comprising the scale-dependent occurrences of local maxima. The Matlab program also determines the upper and lower envelopes by interpolating between the peaks and troughs and calculates the average value of these envelopes as the instantaneous mean. For the interpolation, a piecewise cubic Hermite interpolating polynomial was used in order to ensure a smooth determination of the envelopes[2, 3]. Raw amplitude was calculated as an instantaneous measure of the difference between the upper and lower envelopes. This value was adjusted to a percent value of the mean to take into account differences in vessel size or brightness. For frequency (f) assessment, the number of peaks (N) in the selected interval was determined using AMPD and then the mean value of time intervals between consecutive peaks (ΔN) was calculated. Finally, the frequency was determined according to $f=1/\Delta N$. Quantifications of the median percent amplitude and frequency in contractions per min were performed for the periods excluding the first and last 15 s of the movies. The exclusion of the beginning and end of the time series was performed since the applied peak-detection algorithm needed the beginning and end of the time series for the auto-tuning of parameters while not giving peak information for these time intervals.

The code is as follows:

(1) Main function:

```
function [ResultsAll,Frequency] = AMPDAnalysis_ LymphaticVesselContractility (x,Fs,w)
```

```
%_______________________________________________________________________________
% Software to quantify the lymphatic vessel contractility based on
% optical measurements. For the peak detection the AMPD method is used [1].

% [1] Scholkmann et al. (2012). An Efficient Algorithm for Automatic Peak
% Detection in Noisy Periodic and Quasi-Periodic Signals. Algorithms 2012,
% 5(4), 588-603

% INPUT
%   x: Input signal (intensity or diameter values)
%   Fs: sampling frequency ([Hz])
%   w: window length of the moving average filter (w > 2 must be satisfied)

% OUTPUT
%   ResultsAll: this matrix contains all calculated values:
%       Column 1: Time
%        Column 2: Inst. amplitude, based on the raw signal
%       Column 3: Inst. frequency, based on the raw signal
%       Column 4: Inst. mean, based on the raw signal
%       Column 5: Inst. amplitude, based on the filtered signal
%       Column 6: Inst. frequency, based on the filtered signal
%       Column 7: Inst. mean, based on the filtered signal
%   Frequency: mean frequency value calculated with different approaches
%       Column 1: based on raw signal and MESA spectral analysis
%       Column 2: based on raw signal and peak counting
%       Column 3: based on filtered signal and MESA spectral analysis
%       Column 4: based on filtered signal and peak counting

% EXAMPLE
% ResultsAll,Frequency] =
% AMPDAnalysis_ LymphaticVesselContractility (data(:,6),2.5,50);
% -> Fs: 2.5 Hz, w: 50

% (1) Calculate the characteristics with the raw signal (x)
% (1.1) Peak detection
[M1.raw,M_r1.raw,lambda1.raw,p1.raw] = AMPD(x,100,0);
[M2.raw,M_r2.raw,lambda2.raw,p2.raw] = AMPD(-x,100,0);

% (1.2) Envelopes
env_up.raw = interp1(p1.raw, x(p1.raw), [1:length(x)]', 'pchip', 'extrap');
env_down.raw = interp1(p2.raw, x(p2.raw), [1:length(x)]', 'pchip', 'extrap');

% (1.3) Instantaneous mean and frequency
inst_mean.raw = env_down.raw+(env_up.raw-env_down.raw)./2;
inst_ampl.raw = env_up.raw-env_down.raw;
inst_freq_raw.raw = diff(sort([p1.raw,p2.raw]))/2;
inst_freq.raw = interpft(inst_freq_raw.raw,length(x));
inst_freq.raw = inst_freq.raw';

TSc = (Fs*60);
```

```
time = [1:length(x)]/TSc;
inst_freq.raw = (1./inst_freq.raw)*60/2;
mean(inst_freq.raw)

All.raw = [inst_ampl.raw,inst_freq.raw,inst_mean.raw];
[s, FreqAll.raw] = size(p1.raw);
FreqAll.raw = FreqAll.raw/(max(time)*60);

% (1.4) Spectral analysis
[PD.raw,f.raw] = MESA_FFT(x,Fs*60);

% (2) Calculate the characteristics with the detrended signal (xd)
% (2.1) Detrending
xd = (x-smooth(x,w))+1000;

% (2.2) Peak detection
[M1.filt,M_r1.filt,lambda1.filt,p1.filt] = AMPD(xd,100,0);
[M2.filt,M_r2.filt,lambda2.filt,p2.filt] = AMPD(-xd,100,0);

% (2.3) Envelopes
env_up.filt = interp1(p1.filt, xd(p1.filt), [1:length(xd)]', 'pchip', 'extrap');
env_down.filt = interp1(p2.filt, xd(p2.filt), [1:length(xd)]', 'pchip', 'extrap');

% (2.4) Instantaneous mean and frequency
inst_mean.filt = env_down.filt+(env_up.filt-env_down.filt)./2;
inst_ampl.filt = env_up.filt-env_down.filt;
inst_freq_raw.filt = diff(sort([p1.filt,p2.filt]))/2;
inst_freq.filt = interpft(inst_freq_raw.filt,length(xd));
inst_freq.filt = inst_freq.filt';

TSc = (Fs*60);
time = [1:length(xd)]/TSc;
inst_freq.filt = (1./inst_freq.filt)*60/2;
mean(inst_freq.filt)

All.filt = [inst_ampl.filt,inst_freq.filt,inst_mean.filt];
[s, FreqAll.filt] = size(p1.filt);
FreqAll.filt = FreqAll.filt/(max(time)*60);

% (2.5) Spectral analysis
[PD.filt,f.filt] = MESA_FFT(xd,Fs*60);

%________________________________________________________________________
close all
set(0,'defaultAxesFontName', 'Arial')
scrsz = get(0,'ScreenSize');
figure('Position',[0 0 scrsz(3) scrsz(4)]);
set(gcf, 'color', 'w')
set(gcf,'defaultaxesfontsize',12)

subplot(2,4,1)
```

```
plot(time,x,'k')
hold on; plot(p1.raw/TSc,x(p1.raw),'or','linewidth',2)
title(char('Input signal, peaks, envelopes','    instantaneous mean'),'fontsize',15);
xlabel 'Time [min]'

hold on
plot(time,env_up.raw,'r')
hold on
hold on; plot(p2.raw/TSc,x(p2.raw),'or','linewidth',2)
hold on
plot(time,env_down.raw,'r')
hold on
plot(time,inst_mean.raw,'b','linewidth',2)
axis tight

subplot(2,4,2)
plot(time,inst_ampl.raw,'linewidth',2)
axis tight
ylim([min(inst_ampl.raw)-
0.5*min(inst_ampl.raw),max(inst_ampl.raw)+0.5*max(inst_ampl.raw)])
xlabel 'Time [min]'
title('Instantaneous amplitude','fontsize',15);

subplot(2,4,3)
plot(time,inst_freq.raw,'linewidth',2)
axis tight
ylim([min(inst_freq.raw)-0.5*min(inst_freq.raw),max(inst_freq.raw)+0.5*max(inst_freq.raw)])
xlabel 'Time [min]'
title('Instantaneous frequency','fontsize',15);

[a.raw,b.raw] = max(PD.raw.MESA);

Fmax.raw = f.raw.MESA/60;
Fmax.raw = Fmax.raw(b.raw)

subplot(2,4,4)
plot(f.raw.MESA/60,PD.raw.MESA)
axis tight
title( sprintf( '%s: %d', 'Freq', Fmax.raw,' Hz'));
xlabel 'Frequency [Hz]'

%_____

subplot(2,4,5)
plot(time,xd-1000,'k')
hold on; plot(p1.filt/TSc,xd(p1.filt)-1000,'or','linewidth',2)
title(char('Filtered signal, peaks, envelopes','    instantaneous mean'),'fontsize',15);
xlabel 'Time [min]'

hold on
plot(time,env_up.filt-1000,'r')
```

```
hold on
hold on; plot(p2.filt/TSc,xd(p2.filt)-1000,'or','linewidth',2)
hold on
plot(time,env_down.filt-1000,'r')
hold on
plot(time,inst_mean.filt-1000,'b','linewidth',2)
axis tight

subplot(2,4,6)
plot(time,inst_ampl.filt,'linewidth',2)
axis tight
ylim([min(inst_ampl.filt)-0.5*min(inst_ampl.filt),max(inst_ampl.filt)+0.5*max(inst_ampl.filt)])
xlabel 'Time [min]'
title('Instantaneous amplitude','fontsize',15);

subplot(2,4,7)
plot(time,inst_freq.filt,'linewidth',2)
axis tight
ylim([min(inst_freq.filt)-0.5*min(inst_freq.filt),max(inst_freq.filt)+0.5*max(inst_freq.filt)])
xlabel 'Time [min]'
title('Instantaneous frequency','fontsize',15);

[a.filt,b.filt] = max(PD.filt.MESA);

Fmax.filt = f.filt.MESA/60;
Fmax.filt = Fmax.filt(b.raw)

subplot(2,4,8)
plot(f.filt.MESA/60,PD.filt.MESA)
axis tight
title( sprintf( '%s: %d', 'Freq', Fmax.raw,' Hz'));
xlabel 'Frequency [Hz]'

%_____

 % Create a matrix with all the calculated values:

ResultsAll =
[time',inst_ampl.raw,inst_freq.raw,inst_mean.raw,inst_ampl.filt,inst_freq.filt,inst_mean.filt];
Freq1 = Fmax.raw;
Freq2 = FreqAll.raw;
Freq3 = Fmax.filt;
Freq4 = FreqAll.filt;
Frequency(1,:) = [Freq1,Freq2,Freq3,Freq4];
Frequency(2,:) = [60*Freq1,60*Freq2,60*Freq3,60*Freq4];
%Frequency = Frequency';
```

(2) Subfunction 1

```
function [PD,f] = MESA_FFT(x,Fs)

% Function to calulate the frequency spectrum using the traditional
% aproach (FFT) and based on the Maximum Entropy Spectral Analysis (MESA)
% SUBFUNTIONS:
% mesa.m
% PowerSpectrum

% INPUT
%   x: Input
%   Fs: sampling frequency (Hz)

% OUTPUT
%
%_______________________________________________________________________

order = round(length(x)/2);
x = detrend(x);

% FFT
[PD.FFT,f.FFT] = PowerSpectrum(x,Fs,10000);

% MESA
n = 10;
[PD.MESA] = mesa(x,order,n*Fs);
f.MESA = [1:length(PD.MESA)]/(2*n);




function [p] = mesa(x,m,nfreq)
% SYNTAX: p = mesa(x,m,nfreq);
% For a vector x, this function calculates a maximum-entropy spectrum
% of order m. The spectral estimate is returned in the vector p, which
% has nfreq points linearly spaced in the Nyquist frequency interval 0-.5.
% The psd is normalized such that the mean square value of x equals the
% integral of p from -.5 to .5, so sum(x.^2)/N ~= sum(p)/nfreq.
% Mesa is based on the Burg algorithm, as described in Numerical Recipes
% and implemented in their memcof and evlmem subroutines.
%
% Written by Eric Breitenberger       Version 5/24/95
% Please send comments and suggestions to eric@gi.alaska.edu
%http://www.atmos.ucla.edu/tcd/ssa/matlab/ssa_matlab.m


if min(size(x))>1, error('Row or column vectors only!'), end
[n,c]=size(x);
if c>n, x=x'; n=c; end  % x is now a column vector of size n
x=x-mean(x);  % center the series
```

```
% set up workspace column vectors
wk1=zeros(n,1);
wk2=zeros(n,1);
wk3=zeros(n,1);
wkm=zeros(m,1);
ak=zeros(m,1);

% initialize
a0=sum(x.^2)/n;
wk1(1)=x(1);
wk2(n-1)=x(n);
wk1(2:n-1)=x(2:n-1);
wk2(1:n-2)=x(2:n-1);

% Now calculate a0 and ak via recursion
for k=1:m
  pneum=0;
  denom=0;
  pneum=sum(wk1(1:n-k).*wk2(1:n-k));
  denom=sum(wk1(1:n-k).^2+wk2(1:n-k).^2);
  ak(k)=2.*pneum/denom;
  a0=a0*(1.-ak(k).^2);
  if k>1
    ak(1:k-1)=wkm(1:k-1)-ak(k)*wkm(k-1:-1:1);
  end
  if k==m, break, end
  wkm(1:k)=ak(1:k);
  wk3=wk1;
  wk1(1:n-k-1)=wk1(1:n-k-1)-wkm(k)*wk2(1:n-k-1);
  wk2(1:n-k-1)=wk2(2:n-k)-wkm(k)*wk3(2:n-k);
end

% The coefficients a0 and ak have been calculated, now use these
% to evaluate the psd at nfreq frequencies (eqn. 12.8.4 Num. Rec.).
% By changing how p is initialized, the spacing in f can be changed.
% For example, one could use logspace instead of linspace.

p=2*pi*linspace(0,.5,nfreq);
fc=cos(p);
fs=sin(p);
lc=ones(1,nfreq);     % initialize 'last' cosine and sine
ls=zeros(1,nfreq);
temp=zeros(1,nfreq);
sc=ones(1,nfreq);     % initialize sum of cosine and sine terms
ss=zeros(1,nfreq);

% This next loop can be vectorized but memory use goes way up for only
% a small improvement in speed.
for k=1:m
  temp=lc;
```

```
  lc=lc.*fc - ls.*fs;
  ls=ls.*fc + temp.*fs;
  sc=sc-ak(k).*lc;
  ss=ss-ak(k).*ls;
end
p=a0./(sc.^2 + ss.^2);
```

(3) Subfunction 2

```
function [PD,f] = PowerSpectrum(x,fs,w)

% [PD,f,M,PH] = PowerSpectrum(x,fs,w)

% This function computes the power spectrum of a time series x
%-----------------------------------------------------------------------
% INPUT:
%   x         input time series
%   fs        sampling frequency
%   w         Length of the FFT. If the length of x is less than w,
%             x is padded with trailing zeros to length w.
%             If the length of x is greater than w, the sequence x
%             is truncated.

% OUTPUT:
%   PD        Power values (in W/Hz) of the corresponding frequencies (f)
%   f         Frequency values (in Hz) of the corresponding Power values (PD)
%   M         Frequency value of the greatest peak of the spectrum
%   PH        Phase [in degrees]

%_______________________________________________________________________
% By Felix Scholkmann | Biomedical Engineering Laboratory (BORL),
% University Hospital Zürich. Mail: Felix.Scholkmann@gmail.com
%%

x2 = detrend(x);

% (1) Compute the power spectrum
Y = fft(detrend(x2),w)/length(x2);
P = Y.*conj(Y)/length(Y);
f = fs*(0:length(Y)/2)/length(Y);
PD = P(1:1+length(Y)/2);
```

Supplementary References

1. Scholkmann, F., Boss, J. & Wolf, M. An Efficient Algorithm for Automatic Peak Detection in Noisy Periodic and Quasi-Periodic Signals. *Algorithms* **5**, 588 (2012).
2. Fritsch, F.N. & Carlson, R.E. Monotone Piecewise Cubic Interpolation. *Siam Journal on Numerical Analysis* **17**, 238-246 (1980).
3. Kahaner, D., Moler, M. & Nash, S. Numerical Methods and Software (Prentice Hall, 1988).

Supplementary Movie Legends

Supplementary Movie 1 | Infusion of 0.5 µL of 10 µmol/L P20D680 lymphatic tracer into the inguinal lymph node of a 6 week old Prox1-GFP mouse and rapid perfusion of the efferent CLV. Video shows vessel leading toward axillary region of the mouse and alternates with increasing magnification between NIR and GFP visualization of the vessel. Note that the contractility of the vessel and that the valves are easily visualized with Prox1-GFP expression. Video is displayed at 10x normal speed.

Supplementary Movie 2 | Prox1-GFP CLV before and after infusion of lymphatic tracer. Video shows GFP visualization of the same vessel and lymphatic valve before (1st 8 s) and after (2nd 8 s) infusion into the lymph node of P20D680 as in Video 1. Note that the contraction amplitude of the vessel is increased and that the valve function is activated by the infusion. Video is displayed at 10x normal speed.

Supplementary Movie 3 | High magnification imaging of Prox1-GFP CLV contractility. Video at 63x magnification of GFP signal in a 6 week old Prox-GFP mouse of a flank collecting lymphatic vessel after infusion of P20D680. Video is displayed at 10x normal speed.

Supplementary Movie 4 | Dynamics of a profile of a line bisecting a P20D680 perfused CLV during contractility of the vessel. Video at 63x magnification of NIR signal in a 6 week old Prox-GFP mouse of a flank collecting lymphatic vessel after infusion of P20D680. NIR signal counts are displayed on the y-axis and distance in µm are displayed on the x-axis. Note that increases in the brightness of the vessel (increases in the height of the profile) are reflected as

increases in diameter (widening of the profile) indicating the direct relationship between signal intensity and diameter. Video is displayed at 10x normal speed.

Supplementary Movie 5 | Representative video of a P20D680 perfused CLV response to addition of 0.1% DMSO. NIR video at 63x magnification before (up to 2 min on time scale) and after (from 2 to 8 min on time scale) treatment of 40 µL 0.1% DMSO. Video corresponds to the data shown in Figure 4A and is displayed at 30x normal speed.

Supplementary Movie 6 | Representative video of a P20D680 perfused CLV response to addition of 1 µmol/L PGF2α. NIR video at 63x magnification before (up to 2 min on time scale) and after (from 2 to 8 min on time scale) treatment of 40 µL of 1 µmol/L PGF2α. Vessel is the same shown in Video 5 after addition of 0.1% DMSO. Video corresponds to the data shown in Figure 4B and is displayed at 30x normal speed.

Supplementary Movie 7 | Representative video of a P20D680 perfused CLV response to addition of 10 µmol/L PGF2α. NIR video at 63x magnification before (up to 2 min on time scale) and after (from 2 to 8 min on time scale) treatment of 40 µL of 10 µmol/L PGF2α. Video corresponds to the data shown in Figure 4C and is displayed at 30x normal speed.

Supplementary Movie 8 | Representative video of a P20D680 perfused CLV response to addition of 60 µmol/L PGF2α. NIR video at 63x magnification before (up to 2 min on time scale) and after (from 2 to 8 min on time scale) treatment of 40 µL of 60 µmol/L PGF2α. Video corresponds to the data shown in Figure 4D and is displayed at 30x normal speed.

Supplementary Movie 9 | Representative video of a quiescent CLV after P20D680 perfusion and the response to addition of PGF2α. NIR video at 63x magnification before (up to 2 min on time scale) and after (from 2 to 6 min on time scale) treatment of 40 µL of 60 µmol/L PGF2α. Video is displayed at 20x normal speed. Representative of n=5 independent experiments with PGF2α.

Supplementary Movie 10 | Representative video of a non-perfused Prox1-GFP CLV response to addition of 0.1% DMSO. GFP video at 63x magnification before (up to 2 min on

time scale) and after (from 2 to 8 min on time scale) treatment of 40 µL 0.1% DMSO. Video is displayed at 30x normal speed. Representative of n=3 independent experiments.

Supplementary Movie 11 | Representative video of a non-perfused Prox1-GFP CLV response to addition of PGF2α. GFP video at 63x magnification before (up to 2 min on time scale) and after (from 2 to 8 min on time scale) treatment of 40 µL of 60 µmol/L PGF2α. Vessel is the same shown in Video 10 after addition of 0.1% DMSO. Video is displayed at 30x normal speed. Representative of n=6 independent experiments with PGF2α.

Supplementary Movie 12 | Representative video from non-invasive imaging of the lower limb of a mouse before and after treatment with a NO donor. Injection of 5 µL of 20 µmol/L P20D680 was performed intradermally into the paw to visualize CLV network. NIR videos at 15x magnification was acquired at 2.5 frames per second. Video is displayed at 10x normal speed. Representative of n=6 mice with NO donor treatment.
